# Novel Antibacterial Hydrogels Incorporating Quaternary Ammonium Monomethacrylate: A Pilot Study on Swelling Behavior and Biological Analysis

**DOI:** 10.3390/ma19194206

**Published:** 2026-10-02

**Authors:** Patrycja Kula, Alicja Kazek-Kęsik, Hubert Knap, Izabella Ślęzak-Prochazka, Grzegorz Chladek, Izabela Barszczewska-Rybarek

**Affiliations:** 1 Department of Physical Chemistry and Technology of Polymers, Faculty of Chemistry, Silesian University of Technology, Strzody 9 Str., 44-100 Gliwice, Poland; patrycja.kula@polsl.pl; 2 Department of Inorganic Chemistry, Analytical Chemistry and Electrochemistry, Faculty of Chemistry, Silesian University of Technology, B. Krzywoustego 6 Str., 44-100 Gliwice, Poland; alicja.kazek-kesik@polsl.pl (A.K.-K.); hubert.knap@polsl.pl (H.K.); 3 Biotechnology Centre, Silesian University of Technology, B. Krzywoustego 8 Str., 44-100 Gliwice, Poland; izabella.slezak-prochazka@polsl.pl; 4 Department of Systems Biology and Engineering, Faculty of Automatic Control, Electronics and Computer Science, Silesian University of Technology, Akademicka 16 Str., 44-100 Gliwice, Poland; 5 Materials Research Laboratory, Faculty of Mechanical Engineering, Silesian University of Technology, Konarskiego 18A Str., 44-100 Gliwice, Poland; grzegorz.chladek@polsl.pl

**Keywords:** antimicrobial hydrogel, quaternary ammonium monomer, swelling behavior, bacterial adhesion, disk diffusion assay, cytocompatibility

## Abstract

Poly(acrylamide) (PAM) hydrogels offer biomedical potential, but bacterial colonization limits their long-term use. This study examines PAM hydrogels modified with quaternary ammonium monomethacrylate (QAHAMA-10) for antimicrobial properties. Five formulations with QAHAMA-10 to acrylamide (AA) molar ratios ranging from 2.5 to 30 mol.% and crosslinked with 1 mol.% N,N′-methylenebisacrylamide (bis-AA) were evaluated. QAHAMA-10 improved hydrogel stability, especially at 10 mol.%, which showed the best structural integrity and transparency. The degree of swelling (*DS*) decreased with increasing QAHAMA-10 content, from 394.4% (2.5 mol%) to 180.0% (30 mol%), versus 938.4% for the reference. No bacterial growth was detected in suspensions recovered from wells with experimental hydrogels. The hydrogels inhibited *Staphylococcus aureus ATCC 25923*, with inhibition zones ranging from 16.2 to 23.1 mm depending on the QAHAMA-10 content, while the reference showed no inhibition. In contrast, no activity was observed against *Escherichia coli ATCC 25922* in the disk diffusion assay. Alamar Blue reduction in fibroblast L929 cells decreased with higher QAHAMA-10 content, ranging from 70.8% to 27.7% after 24 h and 74.4% to 26.1% after 48 h, compared to 78.1% and 94.2% for the reference. Overall, 2.5 mol.% QAHAMA-10 offered the best balance of anti-*S. aureus* activity and cytocompatibility, while 10 mol.% provided optimal physicochemical properties.

## 1. Introduction

**Scheme 1:**
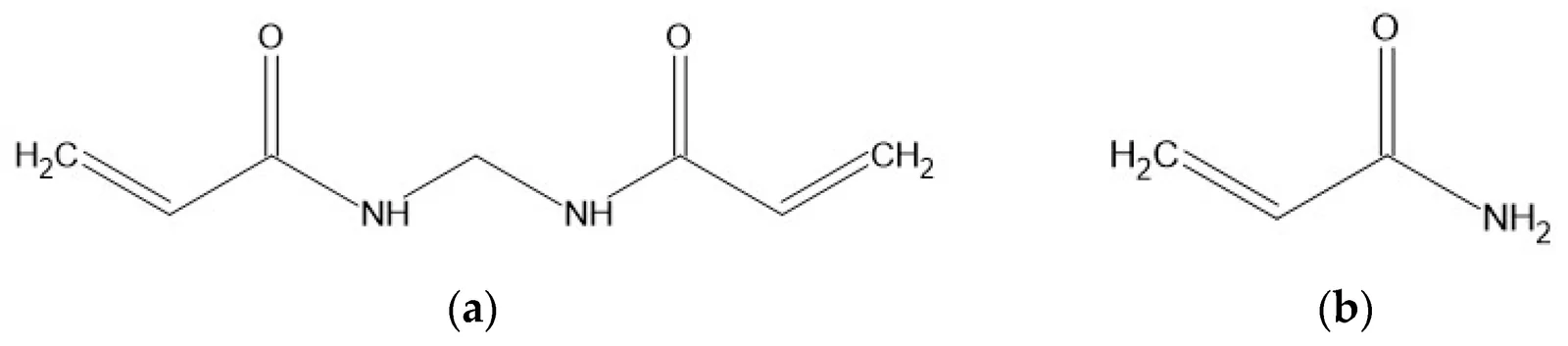
Chemical structures of (**a**) N,N′-methylenebisacrylamide and (**b**) acrylamide.

Poly(acrylamide) (PAM) hydrogels based on acrylamide (AA) and N,N′-methylenebisacrylamide (bis-AA) crosslinker (Scheme 1) exhibit high water absorption capacity, good mechanical stability, low toxicity, and are easily tunable [1,2,3]. These features make them attractive materials for biomedical applications, such as wound dressings [4], drug delivery systems [5,6], tissue engineering [6], and protein electrophoresis [7], as well as advanced platforms including localized cancer chemo-immunotherapy [8] and biointegrated electronic devices [9]. Additionally, they often serve as model hydrogel systems for studying cell–material interactions and cellular responses [3].

Despite their numerous advantages, a major limitation of PAA hydrogels is susceptibility to bacterial colonization during prolonged tissue contact, which may lead to infections, delayed tissue regeneration, and compromised material performance [10,11]. Consequently, imparting inherent antimicrobial properties to these materials has become an important strategy for improving their safety and clinical efficacy [12]. Various approaches have been developed to address this challenge, ranging from the incorporation of antibiotics, including ciprofloxacin [13], to metal nanoparticles, such as silver nanoparticles [14], and nitric oxide donors [15]. However, the use of physically loaded antimicrobial agents is associated with several limitations, including a gradual loss of activity due to their release, as well as the potential risk of promoting bacterial resistance. In addition, some formulations require external stimuli for activation, further restricting their practical utility [12].

These limitations have prompted the development of alternative hydrogel systems based on polymers with inherent antimicrobial activity, such as chitosan. In its native form, this activity is mediated by the protonation of amino groups (–NH_2_) into positively charged –NH_3_^+^. However, this process is strongly pH-dependent, restricting chitosan’s use under physiological conditions due to insolubility above pH 6.3 [16]. Quaternization is therefore commonly employed to improve chitosan’s solubility and antibacterial activity by introducing permanently positively charged sites into the polymer structure [17]. The resulting quaternary ammonium (QA) groups drive electrostatic interactions with negatively charged microbial membranes, while the hydrophobic alkyl chains penetrate and disrupt the lipid bilayer. This mechanism contributes to the broad antimicrobial activity of QA-based polymers against Gram-positive and Gram-negative bacteria, fungi, and some viruses [18].

Building on this concept, incorporating a QA-bearing monomer directly into the hydrogel structure ensures that the antimicrobial functionality remains an integral part of the polymer. Among the few available reports, a common strategy involves the copolymerization of N-isopropylacrylamide with QA-functionalized methacrylates to impart antimicrobial activity. Within this approach, Raorane et al. [19] developed uncrosslinked, water-soluble copolymers bearing N-alkyl chains that effectively disrupted *E. coli* and *Candida albicans* biofilms. However, their lack of crosslinking precluded shape retention. To achieve structural stability, Wang et al. [20] utilized bis-AA to obtain crosslinked networks, though their application was restricted to thin coatings on polyethersulfone membranes to reduce *S. aureus* and *E. coli* colonization.

**Scheme 2:**
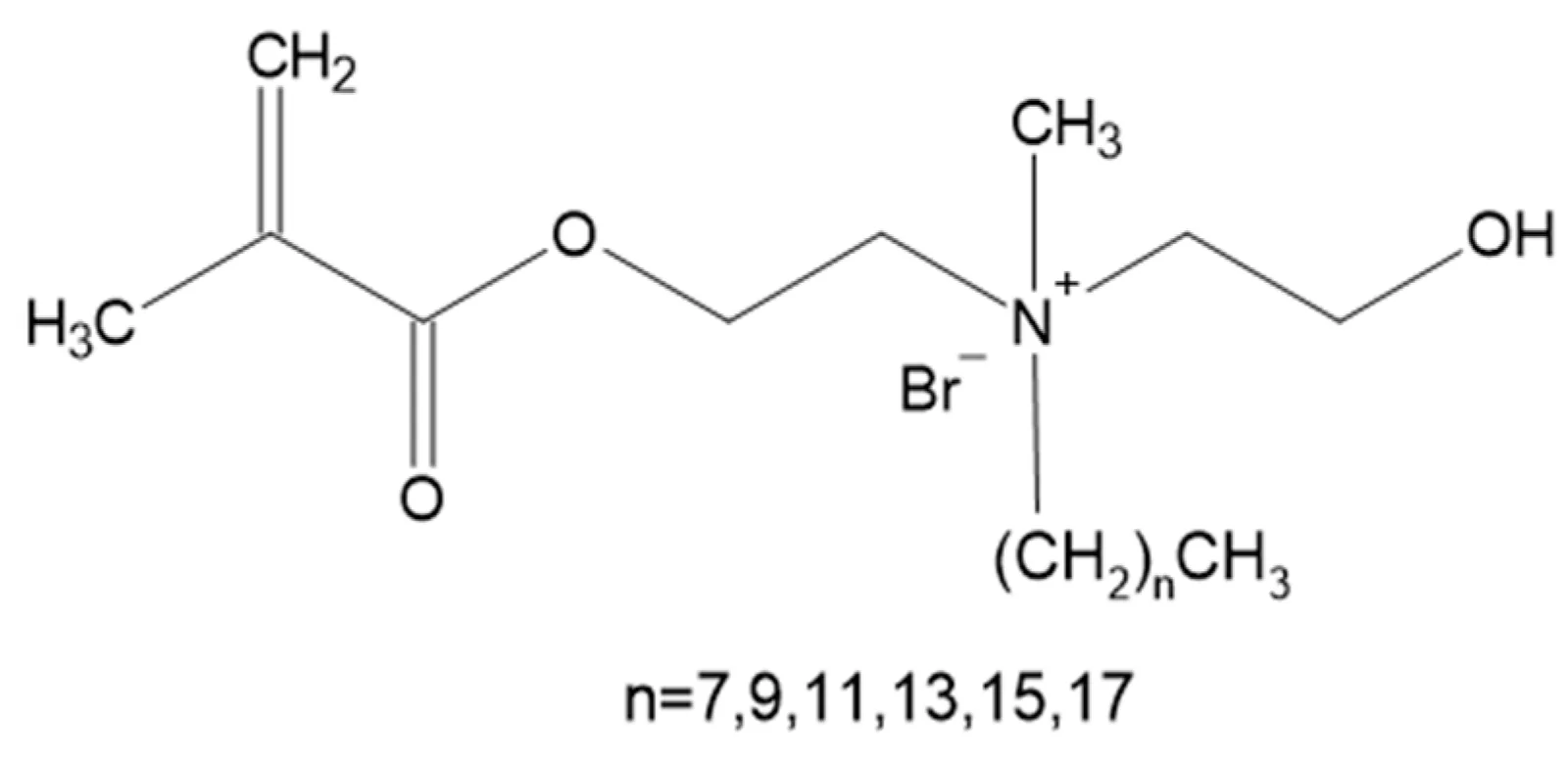
Chemical structure of 2-(methacryloyloxy)ethyl-2-hydroxyethylmethylalkylammonium bromides (QAHAMA-n).

As part of our ongoing research on antimicrobial polymers, our group has developed a series of QA-containing methacrylate monomers, specifically 2-(methacryloyloxy)ethyl-2-hydroxyethylmethylalkylammonium bromides (QAHAMA-n, where n represents the N-alkyl chain length of 8, 10, 12, 14, 16, or 18 carbon atoms, Scheme 2) [21].

Previous studies by Chrószcz-Porębska et al. [22,23] demonstrated that dimethacrylate copolymers functionalized with QAHAMA-n-derived urethane-dimethacrylates (QAUDMAs) exhibited pronounced antibacterial activity against *S. aureus* and *E. coli*, along with antifungal efficacy against *C. albicans*. This biocidal effect depended on the N-alkyl chain length, with the highest activity observed for QAUDMA-8 and QAUDMA-10.

These findings demonstrated the potential of QAHAMA-n-derived structures to provide antimicrobial functionality in polymeric materials and prompted us to explore their application in hydrogel systems.

To the best of our knowledge, the modification of PAM hydrogels through the copolymerization of AA and bis-AA with QAHAMA-n as an antimicrobial component has not been previously reported. The selection of QAHAMA-10 for the present study was based on its favorable combination of antimicrobial efficacy, cytocompatibility, and water sorption capacity, as demonstrated in our previous studies [22,23,24,25]. The polymerizable methacrylate group enables QAHAMA-10 to undergo free-radical copolymerization with AA and bis-AA, while its quaternary ammonium (QA) groups provide contact-active antibacterial functionality.

Therefore, this preliminary study aimed to investigate the effect of QAHAMA-10 concentration on the visual appearance and structural integrity, degree of swelling (*DS*), antibacterial activity, and cytocompatibility of PAM networks.

## 2. Materials and Methods

### 2.1. Chemicals and Reagents

1-bromodecane (BD), N-methyldiethanolamine (MDEA), methyl methacrylate (MMA), deuterated chloroform (CDCl_3_), and tetramethylsilane (TMS) were purchased from Acros Organics (Geel, Belgium). Acrylamide (AA), N,N′-methylenebisacrylamide (bis-AA), potassium periodate (KIO_4_), and phenothiazine (PTZ) were purchased from Sigma-Aldrich (St. Louis, MO, USA). Chloroform, potassium carbonate (K_2_CO_3_), anhydrous magnesium sulfate (MgSO_4_), and toluene were purchased from POCH S.A. (Gliwice, Poland).

### 2.2. QAHAMA-10 Synthesis

**Scheme 3:**
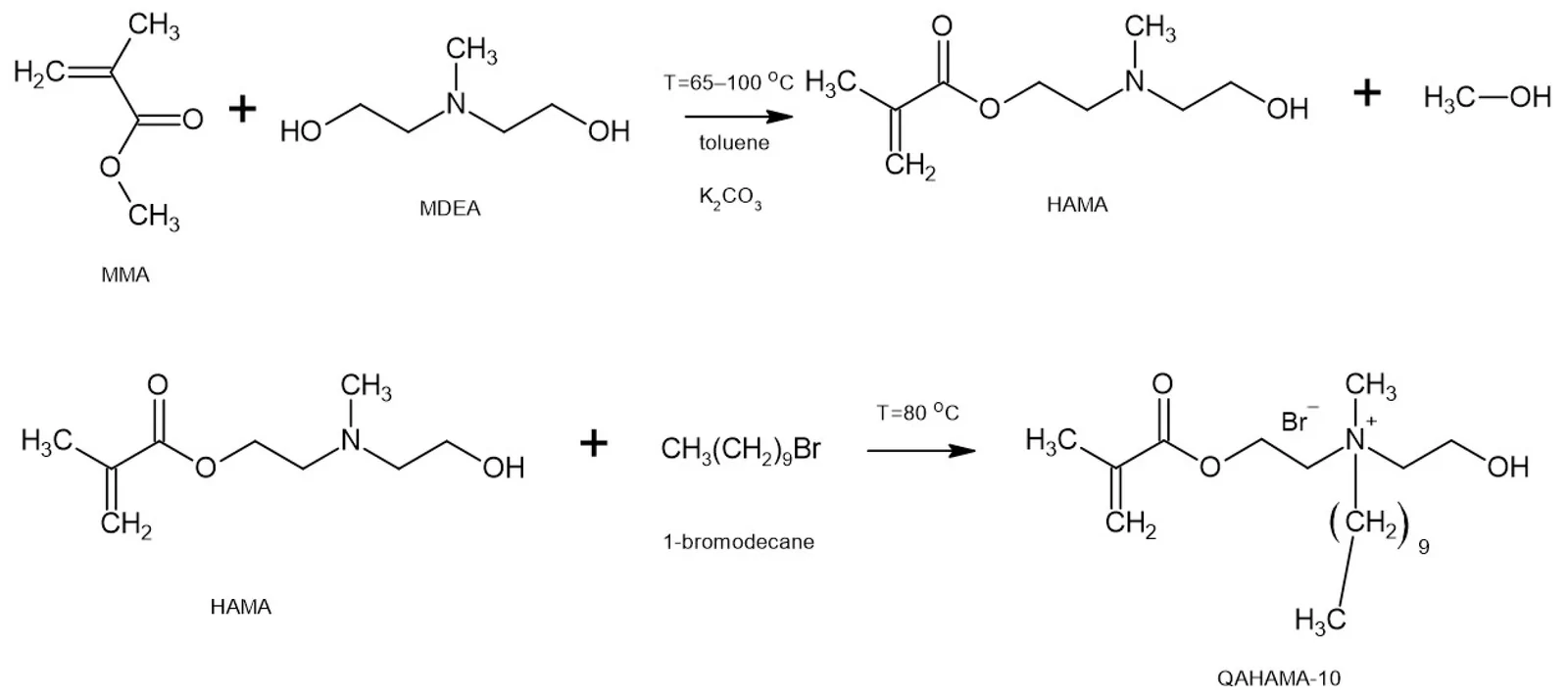
The synthesis route of QAHAMA-10.

The synthesis of QAHAMA-10 was performed according to the procedure developed by Chrószcz et al. [21]. First, MMA (1 mol) was subjected to transesterification with MDEA (0.67 mol) in the presence of K_2_CO_3_ (8 wt%) as a catalyst and PTZ (500 ppm) as a polymerization inhibitor, using toluene as the solvent. The reaction was conducted for 2.5 h, during which the azeotropic fraction was continuously removed, and the temperature at the top of the Vigreux column gradually increased from approximately 65 to 100 °C. After completion of the reaction, the mixture was extracted with water, and the aqueous phase was subsequently extracted with chloroform. The chloroform extract was dried over anhydrous MgSO_4_ and filtered, and the solvent was removed under reduced pressure using a rotary evaporator. The crude HAMA was then purified by vacuum distillation, affording a final yield of approximately 20%. In the next step, HAMA was N-alkylated with BD in the presence of PTZ, and the reaction mixture was stirred at 82 °C for 120 h. The reaction yield was 100% (Scheme 3).

**Figure 1:**
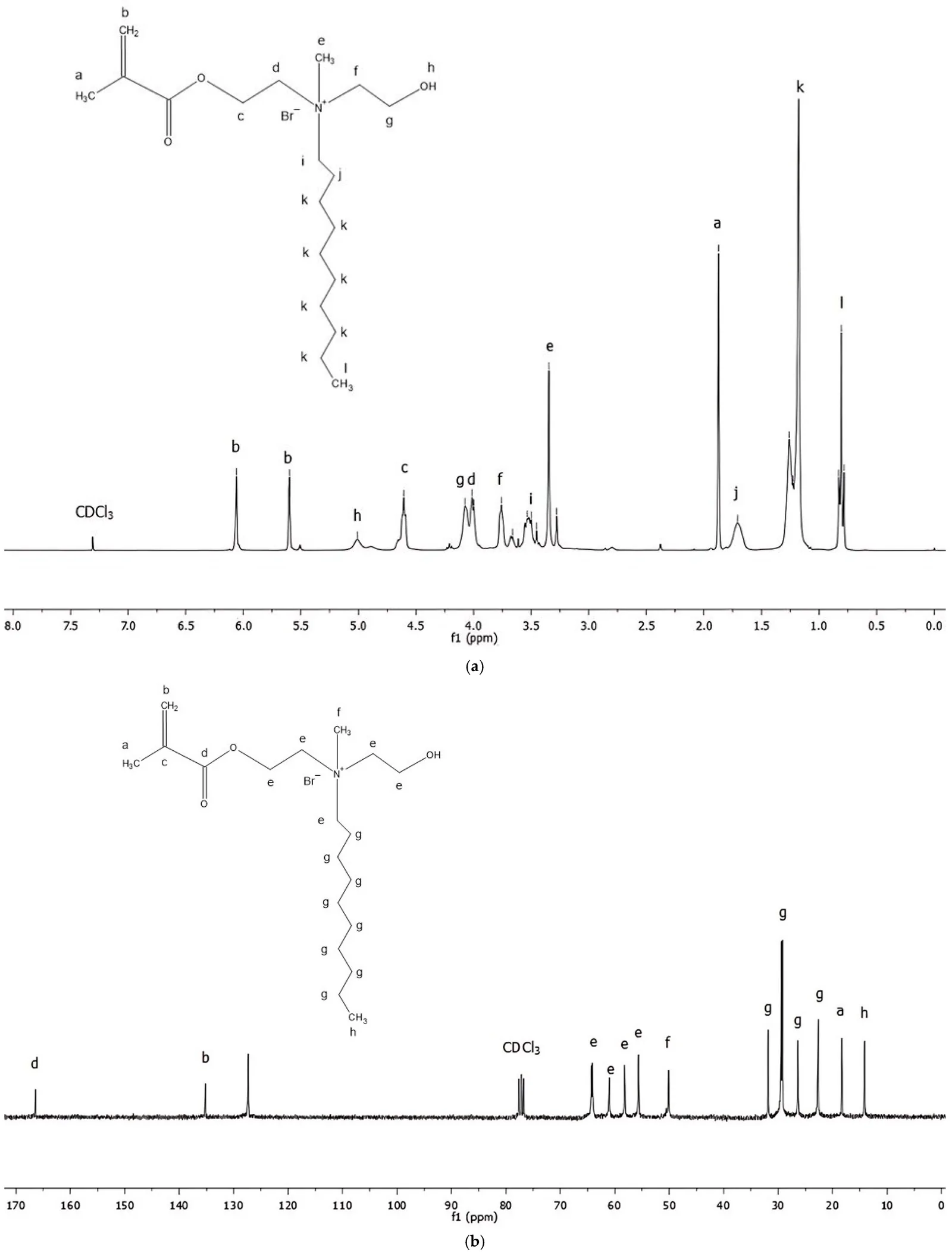
^1^H NMR spectrum (**a**) and ^13^C NMR spectrum (**b**) of QAHAMA-10.

The structure of QAHAMA-10 was confirmed by proton and carbon-13 nuclear magnetic resonance spectroscopy (^1^H and ^13^C NMR) utilizing a 300 MHz instrument (UNITY/INOVA, Varian, Palo Alto, CA, USA). For the ^1^H spectrum, 256 scans were acquired, while the ^13^C spectrum was recorded with 40,000 scans. CDCl_3_ was used as the solvent, and TMS served as the internal standard. The ^1^H and ^13^C NMR spectra, chemical shifts, and corresponding group assignments are summarized in Figure 1 and Table 1 and Table 2.

### 2.3. Hydrogel Preparation

Six hydrogel formulations were prepared and designated as H-0 (control), H-I, H-II, H-III, H-IV, and H-V. The experimental formulations contained QAHAMA-10, AA, bis-AA, and 10 mL of a 0.15 wt.% aqueous KIO_4_ solution, whereas the control formulation (H-0) was prepared without QAHAMA-10. Throughout all formulations, the bis-AA content was maintained at approximately 1 mol.% relative to the total molar amount of AA, QAHAMA-10, and bis-AA, while the molar ratio of AA and QAHAMA-10 was varied. The detailed compositions are shown in Table 3.

**Figure 2:**
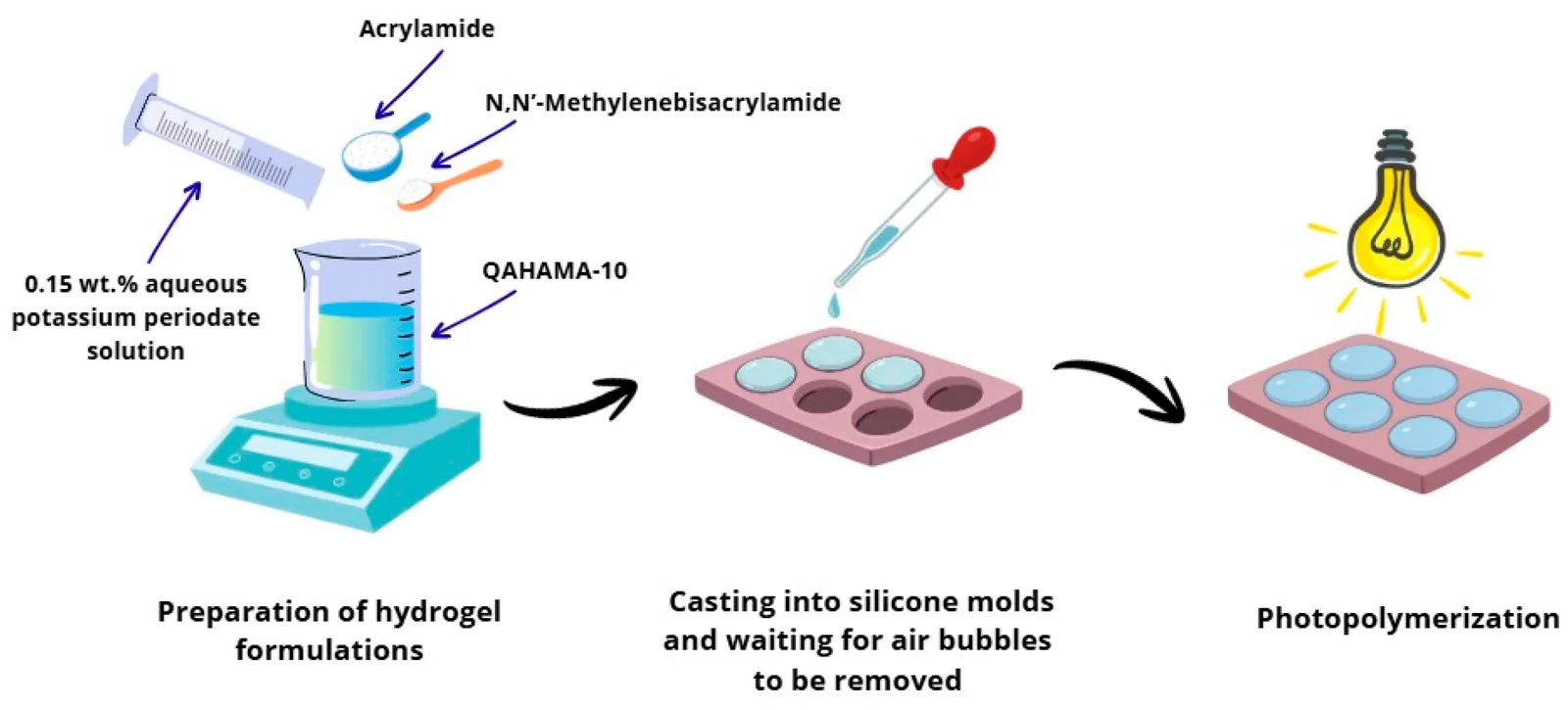
Procedure for formulating and curing experimental hydrogel compositions.

All components of the liquid hydrogel formulations were mixed using a magnetic stirrer at room temperature (Figure 2). The homogeneous mixtures were cast into silicone molds to obtain disc-shaped specimens measuring 10 × 2 mm (diameter × thickness). The formulations were allowed to stand for 30 min to release air bubbles. To initiate photopolymerization, the specimens were irradiated at room temperature for 30 min at a fixed working distance of 15 cm utilizing a broad-spectrum UV–VIS lamp (Ultra Vitalux 300 W, OSRAM, Munich, Germany, diameter of 127 mm, providing a radiated power of 3.0 W in the UV-B range (280–315 nm) and 13.6 W in the UV-A range (315–400 nm) [26].

### 2.4. Qualitative Assessment

The hydrogel specimens were carefully removed from the molds and immersed in 200 mL of distilled water at room temperature for 7 days to reach swelling equilibrium. To ensure the reliability and consistency of the qualitative assessment, a panel of three independent evaluators performed the evaluation through visual inspection and gentle tactile manipulation. Prior to the assessment, the evaluators aligned the evaluation criteria. Specifically, the hydrogels were assessed based on (i) optical appearance (classified as transparent or opaque and homogeneous or heterogeneous); (ii) structural integrity during swelling (monitoring for signs of disintegration); and (iii) fracture susceptibility (recording the presence of cracks or brittleness). A total of five specimens of each hydrogel were evaluated, and the final consensus descriptions were recorded.

### 2.5. Degree of Swelling

Five specimens of each hydrogel were immersed in 200 mL of distilled water at room temperature for 7 days, after which the swelling was measured. Afterward, the samples were removed and gently blotted with filter paper to eliminate excess surface water. The mass of the swollen hydrogel (*m_s_*) was measured using an analytical balance (XP Balance, Mettler Toledo, Greifensee, Switzerland) with an accuracy of 0.0001 g. The specimens were subsequently lyophilized using a benchtop freeze dryer (FreeZone 2.5 Liter, Labconco, Kansas City, MO, USA) and reweighed to determine the mass of the dried polymer (*m_d_*). Finally, the degree of swelling (*DS*) was calculated according to the following equation:

(1) $$\mathrm{DS}\;\left(\%\right)=\frac{m_{s}-m_{d}}{m_{d}}\times 100\%$$

where

*m_s_*—the mass of the swollen hydrogel sample;

*m_d_*—the mass of the dried hydrogel sample.

### 2.6. Antibacterial Activity

The antibacterial activity of the hydrogels was evaluated against *S. aureus* (ATCC 25923) and *E. coli* (ATCC 25922) using bacterial suspensions at an initial concentration of approximately 5 × 10^8^ CFU/mL. Three hydrogel samples (*n* = 3) of each formulation were individually placed into the wells of a 24-well culture plate (Biologix, Shandong, China) and immersed in 1 mL of the bacterial suspension. The plates were incubated at 37 °C for 18 h. Following incubation, the hydrogel samples were removed from the wells, and the remaining bacterial suspension was collected. A 100 µL aliquot of the collected suspension was directly spread, without prior dilution, onto Müller–Hinton agar plates (Diag-Med, Warsaw, Poland) using a sterile spreader. The plates were subsequently incubated at 37 °C for 18 h. Bacterial viability was assessed qualitatively based on the presence or absence of visible bacterial growth on the agar plates. Extensive bacterial growth indicated lower antibacterial activity, whereas absent growth indicated a stronger antibacterial effect. In cases of confluent bacterial growth, the initial bacterial concentration was used as the reported CFU/mL value. For the disk diffusion test, bacterial suspensions were evenly inoculated onto the entire surface of Müller–Hinton agar plates using a sterile cotton swab. Three hydrogel samples (*n* = 3) of each formulation were then placed on the inoculated agar surface. The plates were incubated at 37 °C for 18 h. After incubation, the inhibition zone diameter (*IZD*) surrounding each hydrogel sample was measured transversely through the center of the sample using a caliper.

### 2.7. Alamar Blue Assay in L929 Fibroblast Cells

Three hydrogel samples (*n* = 3) with a total surface area of 7.54 cm^2^ of each formulation were sterilized by exposure to UV light for 45 min in a laminar flow hood. Subsequently, each sample was immersed in 5 mL of Dulbecco’s Modified Eagle Medium (DMEM, PAN-Biotech, Germany) containing 4.5 g/L glucose, L-glutamine, and 3.7 g/L NaHCO_3_, resulting in a surface-area-to-extraction ratio amounting to 1.51 cm^2^/mL (1.51 cm^2^ of area to 1 mL of culture medium). The samples were incubated at 37 °C for 72 h in a humidified atmosphere containing 5% CO_2_ to obtain hydrogel extracts (according to ISO 10993-5:2009 [27]). After incubation, the extracts were filtered through sterile 0.22 µm nylon syringe filters.

Alamar Blue reduction was evaluated using L929 mouse fibroblasts (ATCC CCL-1). Cells were cultured in DMEM supplemented with 10% FBS, penicillin/streptomycin, and L-glutamine at 37 °C in a humidified atmosphere containing 5% CO_2_.

The cell suspension containing 5000 cells and 50 µL of the corresponding hydrogel extract (amounting to a 1:1 volume ratio) was added to each well of a 96-well plate. Each hydrogel extract was tested in three independent experiments with three technical replicates. Tissue culture polystyrene (TCPS) was used as the negative control, which consisted of equal parts of 50 μL of cell suspension and 50 µL of cell culture medium, while wells containing culture medium and Alamar Blue reagent without cells served as blanks. The cells were incubated with the hydrogel extracts for 24 and 48 h. Subsequently, 10 µL of Alamar Blue reagent was added to each well, followed by incubation for 4 h. Absorbance was measured at 570 and 600 nm using a Varioskan LUX Multimode Microplate Reader (Thermo Scientific, Waltham, MA, USA).

The percentage reduction in Alamar Blue was calculated according to the following equation:

(2) $$\mathrm{AlamarBlue}\;\mathrm{reduction}\;\%=\frac{100\cdot (\left(O2\cdot A1\right)-(O1\cdot A2))}{\left(R1\cdot N2\right)-(R2\cdot N1)},$$

where

*O*_1_—molar extinction coefficient of oxidized Alamar Blue at 570 nm;

*O*_2_—molar extinction coefficient of oxidized Alamar Blue at 600 nm;

*R*_1_—molar extinction coefficient of reduced Alamar Blue at 570 nm;

*R*_2_—molar extinction coefficient of reduced Alamar Blue at 600 nm;

*A*_1_—absorbance of the experimental sample at 570 nm;

*A*_2_—absorbance of the experimental sample at 600 nm;

*N*_1_—absorbance of the blank sample at 570 nm;

*N*_2_—absorbance of the blank sample at 600 nm.

### 2.8. Statistical Analysis

Statistical analysis was performed using one-way analysis of variance (ANOVA), followed by Tukey’s post hoc test. The results are presented as mean values ± standard deviation (*SD*). Differences were considered statistically significant at *p* ≤ 0.05. All calculations were carried out using Microsoft Excel (version 2608).

## 3. Results

### 3.1. Qualitative Assessment

**Figure 3:**
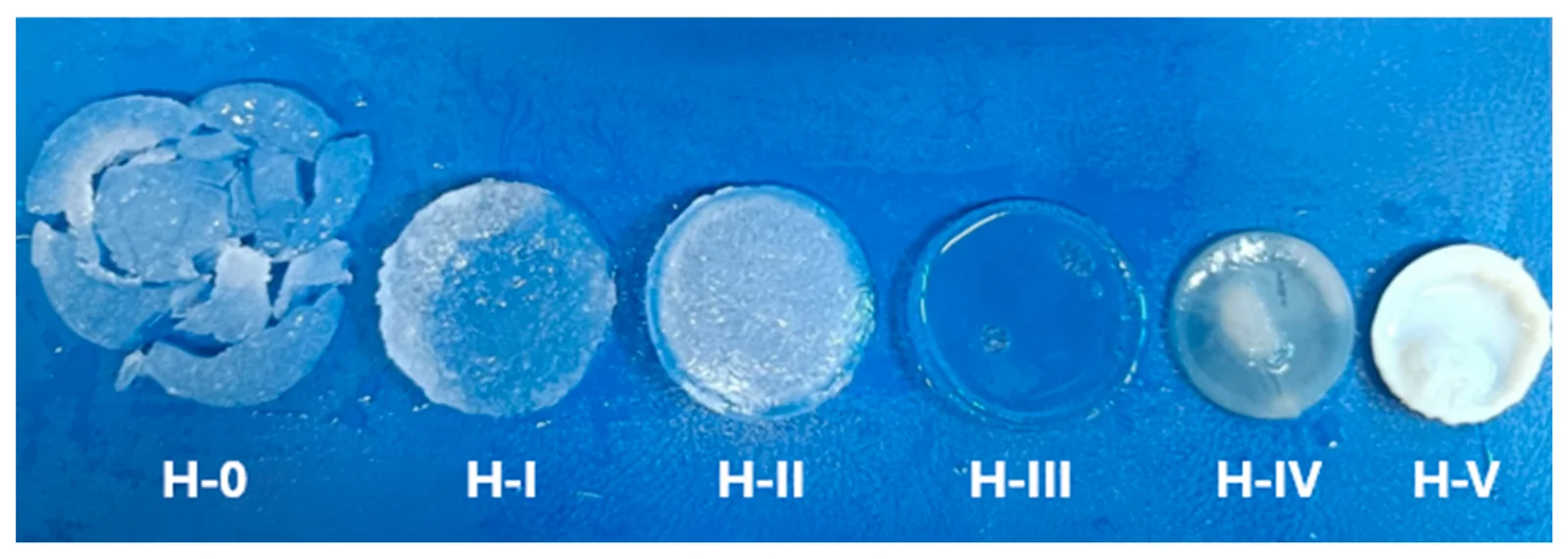
Swollen hydrogel samples.

The macroscopic characteristics of the hydrogels changed significantly after adding QAHAMA-10 (Figure 3, Table 4). The reference hydrogel (H-0), prepared without the antibacterial monomer, was opaque and disintegrated during swelling, which represents an unstable structure. The incorporation of 2.5 mol.% QAHAMA-10 (H-I) substantially improved its structural integrity, preventing disintegration during swelling. However, this hydrogel remained opaque and brittle, reflecting weak overall stability. Increasing the QAHAMA-10 content to 5 mol.% (H-II) further enhanced structural integrity, although moderate brittleness remained, and the hydrogel was still opaque, corresponding to moderate performance. The hydrogel containing 10 mol.% QAHAMA-10 (H-III) exhibited the most favorable overall features, being transparent, highly flexible during handling, and maintaining structural integrity without visible cracks or signs of disintegration, rendering it excellent. Further increasing the QAHAMA-10 concentration did not yield additional improvement. H-IV (20 mol.%) became opaque again but retained good structural integrity and durability. Finally, H-V (30 mol.%) was more brittle than H-IV, though it maintained its shape and flexibility during handling.

### 3.2. Degree of Swelling

**Figure 4:**
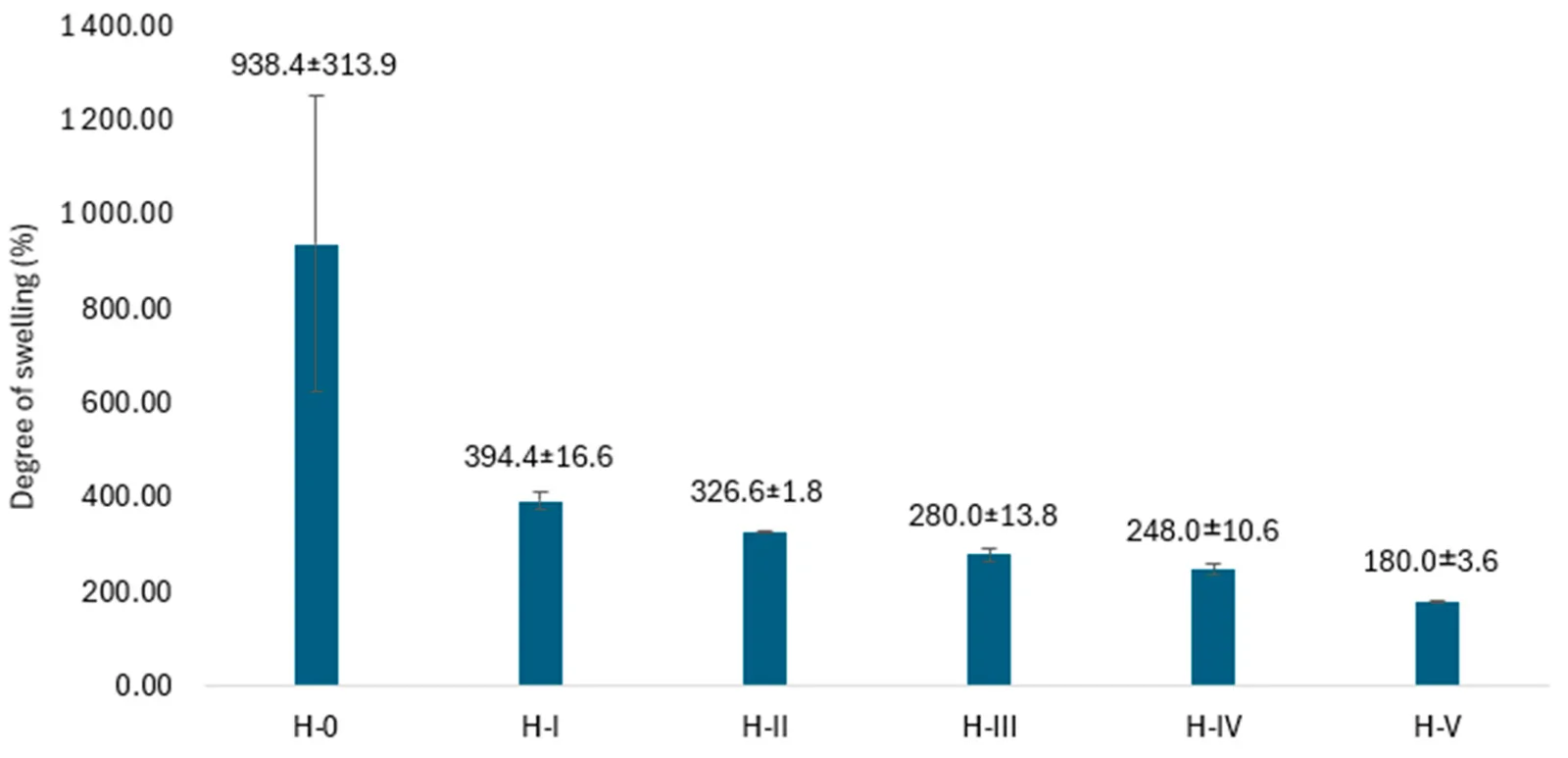
The degree of swelling (*DS*) of hydrogels. All differences were statistically significant (*p* ≤ 0.05).

The swelling behavior of the investigated hydrogels strongly depended on their composition (Figure 4). The reference hydrogel (H-0) exhibited the highest *DS*, reaching 938.4 ± 313.9%. Incorporation of QAHAMA-10 resulted in a progressive decrease in *DS* with increasing QAHAMA-10 content. H-I and H-II exhibited *DS* values of 394.4 ± 16.6% and 326.6 ± 1.8%, respectively, while further decreases were observed for H-III (280.0 ± 13.8%), H-IV (248.0 ± 10.6%), and H-V (180.0 ± 3.6%). Statistical analysis revealed statistically significant differences between the *DS* of all hydrogel formulations.

### 3.3. Antibacterial Activity

**Figure 5:**
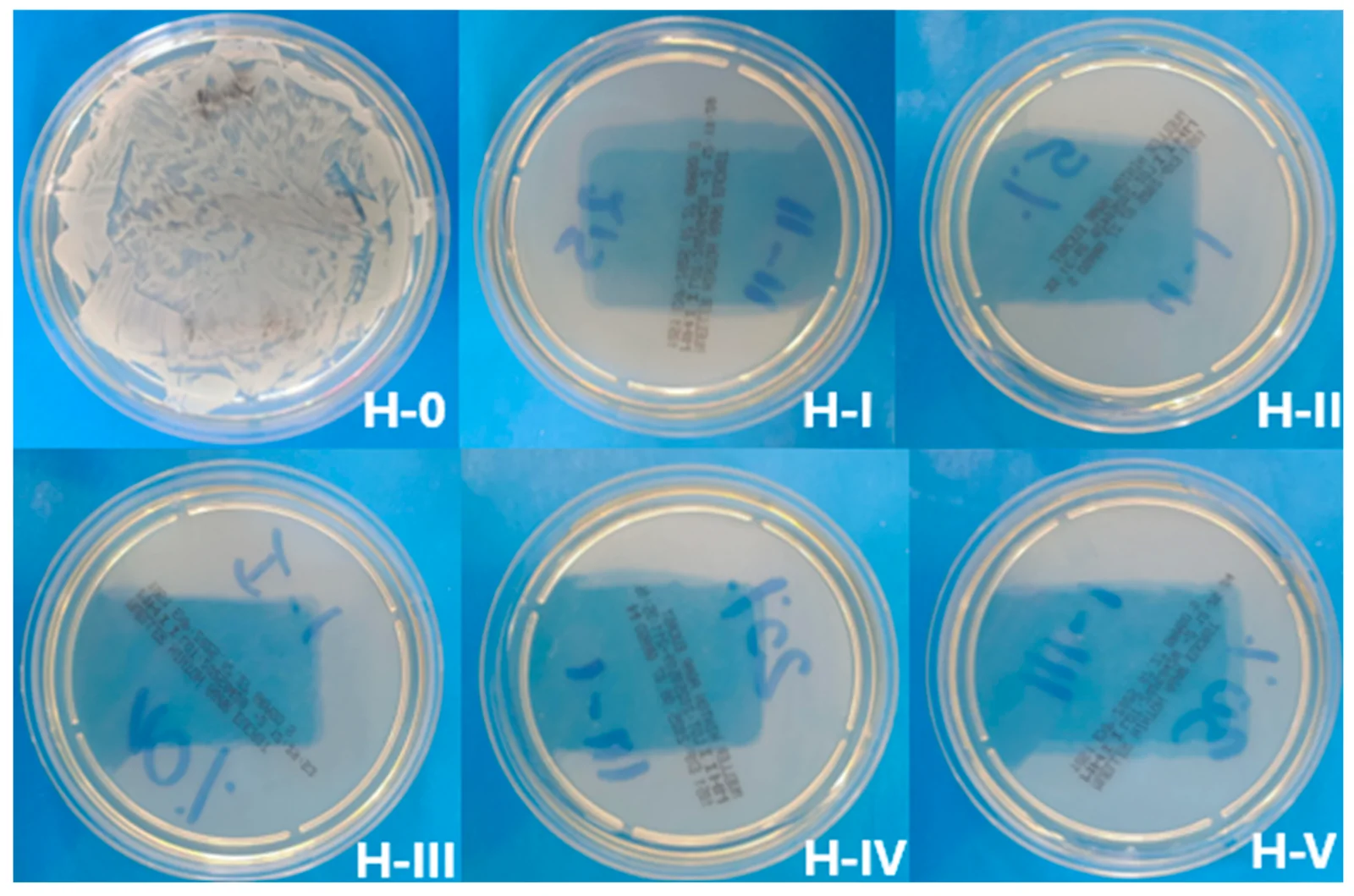
Visual evaluation of *S. aureus ATCC 25923* growth recovered from the hydrogel suspensions.

**Figure 6:**
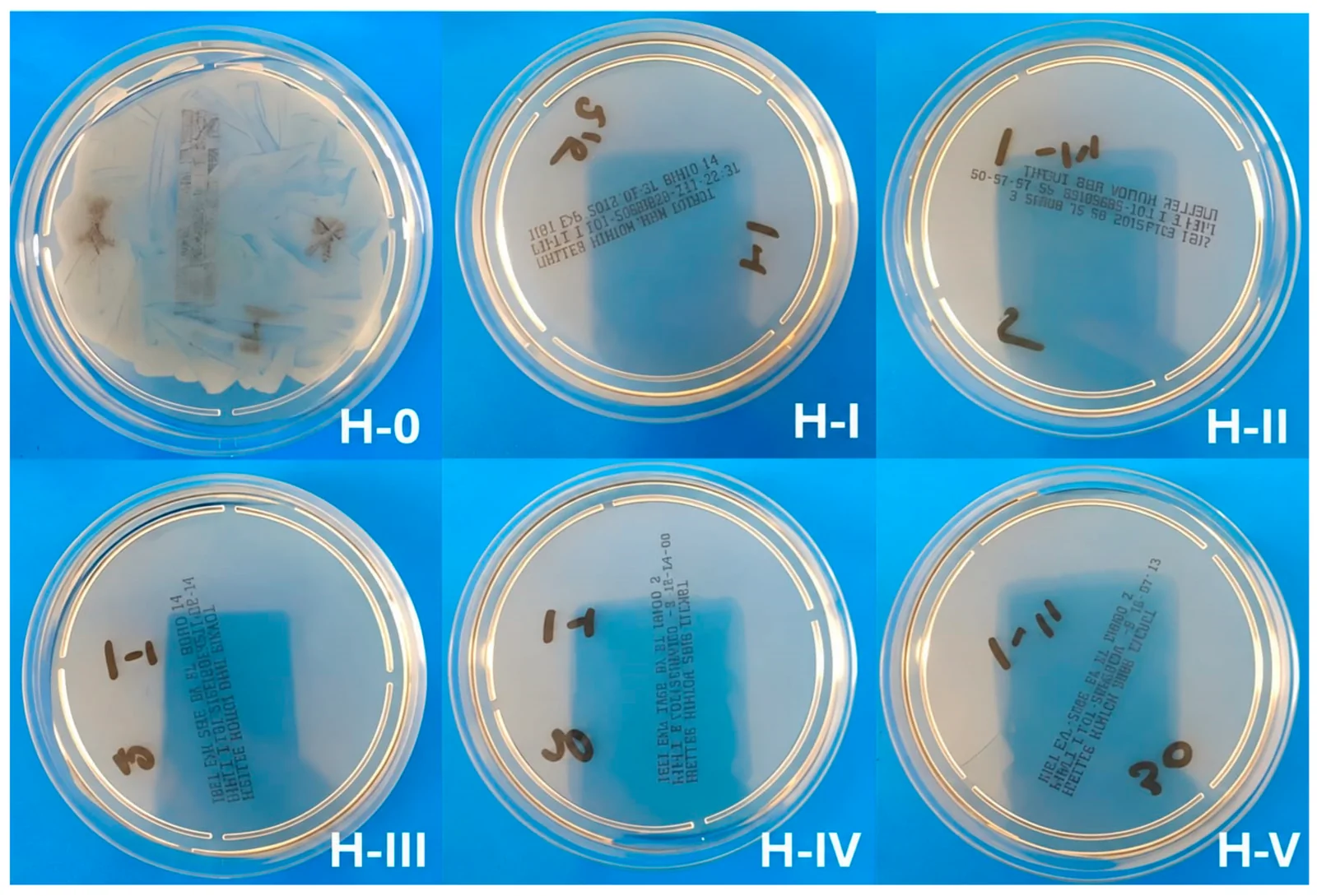
Visual evaluation of *E. coli* ATCC 25922 growth recovered from the hydrogel suspensions.

Antibacterial activity was qualitatively assessed by plating bacteria recovered from the surrounding suspension onto agar plates. No bacterial growth of either tested strain was observed for samples H-I to H-V: *S. aureus* (Figure 5) and *E. coli* (Figure 6). In contrast, bacterial growth recovered from the H-0 control sample was confluent and too dense to count. In cases of confluent bacterial growth, the initial bacterial concentration was reported as 5 × 10^8^ CFU/mL, whereas no bacterial colonies were detected for the experimental hydrogels containing 2.5–30 mol% QAHAMA-10 (0 CFU/mL).

**Figure 7:**
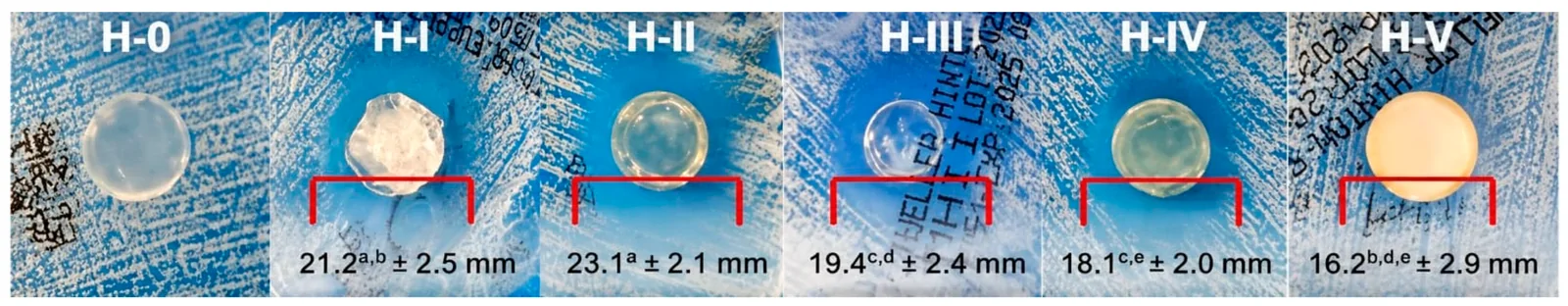
Inhibition zones against *S. aureus ATCC 25923* formed around the hydrogel samples. Values sharing the same superscript letter are not significantly different (*p* > 0.05). All other comparisons are significant (*p* ≤ 0.05).

**Figure 8:**
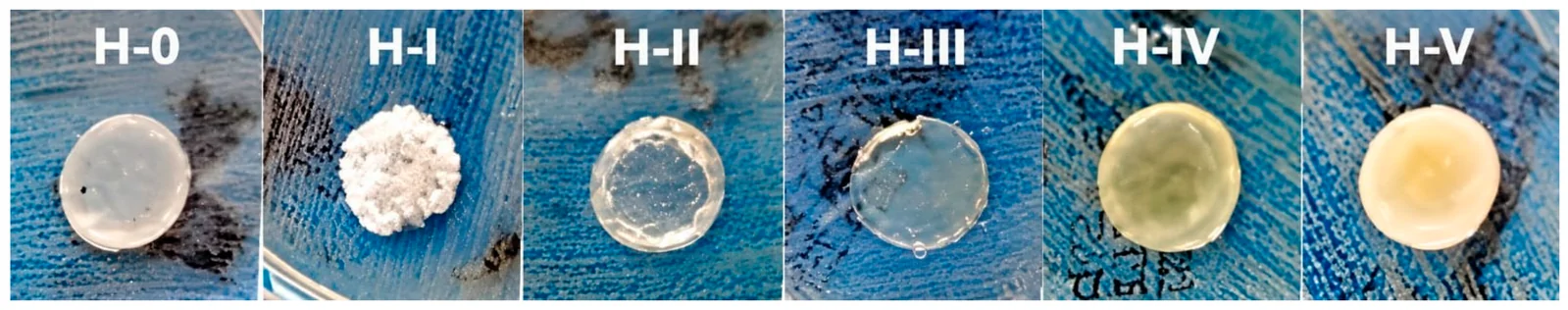
Absence of inhibition zones against *E. coli* ATCC 25922 around the hydrogel samples.

The results of the antimicrobial activity evaluated by the disc diffusion test are presented in Figure 7 and Figure 8.

Against *S. aureus*, no inhibition zone was observed for the reference hydrogel (H-0). In contrast, all QAHAMA-10-modified hydrogels (H-I to H-V) produced measurable inhibition zones (Figure 7), indicating clear antibacterial activity. The highest *IZD* was observed for H-II (23.1 ± 2.1 mm), followed by H-I (21.2 ± 2.5 mm). Lower *IZD* values were recorded for H-III (19.4 ± 2.4 mm), H-IV (18.1 ± 2.0 mm), and H-V (16.2 ± 2.9 mm), indicating a gradual decrease in antibacterial activity as QAHAMA-10 content increased. Statistical analysis revealed no significant difference between H-I and H-II (*p* > 0.05). Similarly, no significant differences were observed between H-I and H-V, H-III and H-IV, H-III and H-V, or H-IV and H-V (*p* > 0.05).

In contrast, no inhibition zones were observed against *E. coli* for any tested hydrogel, including the reference (H-0) and modified samples (H-I to H-V) (Figure 8).

### 3.4. L929 Cell Analysis via Alamar Blue Assay

**Figure 9:**
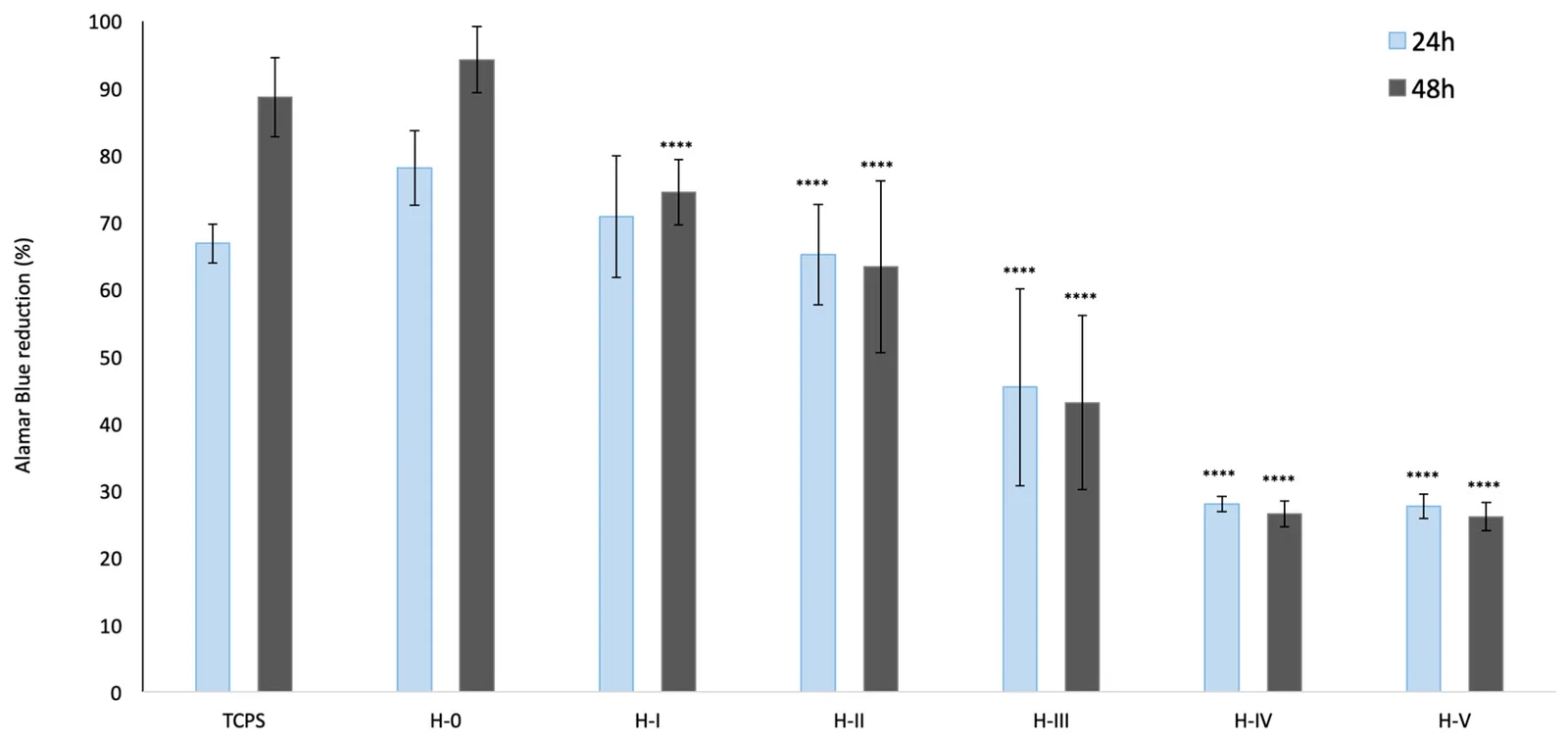
Alamar Blue reduction in the L929 cell line after 24 and 48 h of exposure to hydrogel extracts collected from the HI-HV samples and reference H-0. Statistically significant differences are marked by **** (*p* > 0.0001) (for H-I 24 h, *p* > 0.05) in comparison to the reference H-0 sample. TCPS was used as a negative control.

Figure 9 shows the percentage reduction in Alamar Blue, corresponding to the cell viability of L929 fibroblasts after 24 and 48 h of incubation. TCPS was used as a negative control, whereas H-0 was a reference sample.

After 24 h, the Alamar Blue reduction for the QAHAMA-10-containing hydrogels ranged from 27.7 ± 1.9% for H-V to 70.8 ± 9.1% for H-I. Among the modified hydrogels, H-I exhibited the highest Alamar Blue reduction, whereas a progressive decrease was observed with increasing QAHAMA-10 content. The reference hydrogel H-0 showed the highest Alamar Blue reduction overall (78.1 ± 5.6%), even higher than TCPS.

After 48 h, the Alamar Blue reduction ranged from 26.1 ± 2.1% for H-V to 74.4 ± 4.9% for H-I. Similarly to 24 h, a consistent decrease in Alamar Blue reduction was observed with increasing QAHAMA-10 content, with H-I showing the highest value among the modified hydrogels. The reference hydrogel H-0 again exhibited the highest Alamar Blue reduction overall (94.2 ± 4.9%), and only for this sample and for TCPS, the reduction value increased between the two time points.

## 4. Discussion

The present study showed that copolymerizing QAHAMA-10 with AA and bis-AA produced hydrogels with antibacterial activity while also affecting their optical appearance, structural integrity, handling flexibility, swelling capacity, and biological response. Importantly, these effects were not governed solely by QAHAMA-10 concentration, suggesting a more complex response that may involve the hydrophilic-hydrophobic balance and structural alterations within the hydrogel network.

Macroscopic observations showed that incorporating QAHAMA-10 improved the macroscopic characteristics of hydrogels (Figure 3). The reference H-0 hydrogel was opaque, brittle, and prone to disintegration during swelling. These characteristics may be related to the structural heterogeneity of PAM networks, including local variations in crosslink density and regions enriched in bis-AA arising from differences in AA and bis-AA reactivity during polymerization [3,28,29].

Increasing QAHAMA-10 content altered the hydrogel appearance and structural integrity. H-III, with 10 mol.% QAHAMA-10, demonstrated the best combination of transparency, structural integrity, and durability. While macroscopically this combination of features points toward a well-compatibilized structure, it should be noted that optical transparency alone does not definitively demonstrate a more homogeneous network at the microstructural level [3,29,30]. Further increases in QAHAMA-10 did not enhance these macroscopic characteristics. H-IV became opaque, and H-V exhibited greater brittleness and reduced structural integrity. The return of opacity at higher concentrations may indicate renewed structural heterogeneity, possibly due to changes in compatibility among QAHAMA-10, AA, and bis-AA, or increased influence of hydrophobic N-decyl groups. Such balanced behavior at intermediate compositions is commonly observed in hydrogel systems [31].

The swelling results offered further insight into the impact of QAHAMA-10 on hydrogel properties (Figure 4). H-0 showed a high *DS* of about 938%. Its significant water uptake and likely structural heterogeneity may have caused the observed disintegration during swelling [28,29]. In contrast, adding QAHAMA-10 significantly reduced *DS* and enhanced the hydrogel’s structural integrity. Notably, 2.5 mol.% QAHAMA-10 lowered *DS* by approximately 2.4 times, from 938% to about 394%. The highest QAHAMA-10 concentration reduced *DS* to around 180%.

The reduction in *DS* could be primarily attributed to the effects of QAHAMA-10 incorporation. While the increased hydrophobicity from the QAHAMA-10 monomer is hypothesized to play a key role [32], it is critical to recognize that network swelling is governed by multiple co-existing structural and topological factors. Changes in monomer conversion, the formation of network defects, variations in the soluble fraction, and shifts in the effective crosslink density could also significantly contribute to this trend [33].

Regarding the hydrophobic effects, QAHAMA-10, being a methacrylate, is generally more hydrophobic than AA and bis-AA monomers [32]. Begam et al. [34] observed a similar trend in AA/methyl methacrylate (MMA) hydrogels crosslinked with 1 mol.%. is-AA. Increasing MMA content from 30 to 60 wt.% reduced *DS* from about 651% to 212%, which was attributed to fewer hydrophilic AA units and thus fewer water-binding sites in the network.

In the system investigated in this study, the N-decyl groups of QAHAMA-10 are hypothesized to further reduce water uptake [35,36]. Although QAHAMA-10 contains hydrophilic QA groups, higher concentrations increase the influence of hydrophobic alkyl chains, which might limit water penetration and decrease polymer-chain mobility. As a result, the hydrophilic–hydrophobic balance appears to shift toward lower water uptake as QAHAMA-10 content increases. Abdurrahmanoğlu et al. [37] reported similar effects from hydrophobic associations involving long alkyl chains in PAM-based hydrogels.

The antibacterial results demonstrated that QAHAMA-10 was highly effective when incorporated into the hydrogel network. No viable *S. aureus ATCC 25923* or *E. coli* ATCC 25922 colonies were detected in the bacterial suspension surrounding the QAHAMA-10-containing hydrogels (Figure 5 and Figure 6). In contrast, confluent bacterial growth was observed for the control material H-0. In cases of confluent bacterial growth, the initial bacterial concentration was reported as 5 × 10^8^ CFU/mL, while no bacterial colonies were detected for the experimental hydrogels (2.5–30 mol% QAHAMA-10), and their concentrations were reported as 0 CFU/mL. This demonstrates that the experimental hydrogels exhibited strong antibacterial activity, resulting in no detectable bacterial growth under the tested conditions. It should be noted that the direct-contact assay was performed under static incubation conditions, so bacterial sedimentation during incubation may occur, resulting in the migration of bacteria from the bulk suspension toward the active surface of the samples positioned at the bottom of the wells. On the other hand, the active components may have been released into the surrounding aqueous environment. Both mechanisms may have contributed to the pronounced antibacterial activity of the tested materials, ultimately resulting in complete bacterial reduction.

However, the agar diffusion assay showed that the response depended on both the QAHAMA-10 concentration and the bacterial strain.

For *S. aureus*, the *IZD*s ranged from about 23.1 to 16.2 mm across H-I to H-V, with the highest value observed for H-II containing 5 mol.% QAHAMA-10 (Figure 7). Increasing the QAHAMA-10 concentration therefore did not result in a proportional increase in antibacterial activity. This behavior is consistent with the possibility that diffusion-driven antibacterial effectiveness depends not only on the number of QA groups but also on their accessibility and molecular environment within the polymer network. Since QA groups act through electrostatic interactions with negatively charged bacterial cell surfaces [18,38], at higher concentrations, changes in network organization, crosslink density, or the hydrophilic–hydrophobic balance may restrict the effective diffusion of the active compound into the solid agar medium, thereby limiting the benefit of further increasing QAHAMA-10 content.

The particularly high *IZD* observed for H-II suggests that 5 mol.% QAHAMA-10 may provide a favorable balance between QA group concentration and the network’s physicochemical characteristics. Farah et al. observed a similar concentration-dependent optima for antimicrobial QA-containing polymers, indicating that increasing the amount of antimicrobial groups does not necessarily result in continuously increasing activity [39]. The antibacterial performance of QA-containing polymers has also been associated with the balance between cationic charge and hydrophilic/hydrophobic characteristics [38,40].

The differences in responses between strains can be related to structural differences in their cell envelopes. Gram-negative *E. coli* has an outer membrane rich in lipopolysaccharides, which acts as an additional hydrophobic permeability barrier and restricts interactions with the immobilized QA groups under diffusion conditions. Conversely, the porous peptidoglycan layer of Gram-positive *S. aureus* allowed for effective interaction with the biocidal groups, resulting in the observed zones of inhibition [41,42]. Therefore, the absence of an inhibition zone for *E. coli* does not indicate a lack of antibacterial activity but suggests a predominantly contact-dependent antibacterial effect against Gram-negative strains under the tested conditions (Figure 8).

The assessment of cytocompatibility in L929 fibroblasts revealed a concentration-dependent decrease in Alamar Blue reduction with increasing QAHAMA-10 content (Figure 9). Incorporation of QAHAMA-10 generally reduced cell number relative to H-0, with the lowest values observed for the formulations containing 20 and 30 mol.% QAHAMA-10. Among the modified hydrogels, H-I containing 2.5 mol.% QAHAMA-10 showed the highest cytotoxicity at both 24 and 48 h, whereas H-V containing 30 mol.% exhibited the lowest values. These results indicate that increasing QAHAMA-10 content was associated with a progressive reduction in cell viability.

This behavior may be related to the cationic character of the QA groups. The same electrostatic interactions that contribute to antibacterial activity through interactions with negatively charged bacterial membranes may also promote interactions with mammalian cell membranes and adversely affect cell viability at sufficiently high concentrations [38,43]. The reduction in fibroblast metabolic activity observed with increasing QAHAMA-10 content may therefore reflect the increasing contribution of the QA groups. The consistent decrease in metabolic activity across the investigated concentration range suggests that QAHAMA-10 content was an important factor influencing the cellular response.

The results obtained after 24 and 48 h further showed that this effect persisted throughout the investigated incubation period. Compared with the 24 h results, Alamar Blue reduction increased after 48 h for the reference hydrogel H-0 and H-I, whereas a decrease was observed for H-II to H-V. Despite these time-dependent changes, the overall dose-dependent decrease in metabolic activity was maintained, with H-I exhibiting the highest values and H-V the lowest at both time points.

Taken together, the results indicate that QAHAMA-10 concentration cannot be optimized on the basis of a single hydrogel property. The highest antibacterial properties against *S. aureus* were obtained for H-II with 5 mol.% QAHAMA-10, respectively. In contrast, H-III with 10 mol.% QAHAMA-10 provided the most favorable combination of macroscopic, swelling, and mechanical characteristics while retaining effective antibacterial activity. Its lower fibroblast metabolic activity, however, represents an important limitation for applications involving direct contact with living cells. Within the investigated range, H-III therefore provided the most favorable overall physicochemical characteristics; H-I and H-II showed the highest antibacterial activity against *S. aureus*, whereas H-I exhibited better cytocompatibility among the QAHAMA-10-containing formulations.

These findings suggest that increasing the QAHAMA-10 content above 5 mol.% may not be necessary to achieve effective antibacterial activity. H-I, containing only 2.5 mol.% QAHAMA-10, already exhibited effective antibacterial performance while maintaining the highest cytocompatibility among the modified formulations. Therefore, further development of the hydrogel system should focus on enhancing the structural integrity and durability of the material at lower QAHAMA-10 concentrations, rather than simply increasing the antibacterial monomer content. In particular, future studies should investigate how alternative QA substituents within this concentration range affect the physicochemical characteristics and biological behavior of the hydrogels. Simultaneously, balancing this structural stability with preserved antibacterial activity and cytocompatibility will be critical for further development of these materials.

## 5. Conclusions

QAHAMA-10 can be successfully incorporated into PAM hydrogels to impart effective antibacterial activity. However, increasing its content does not result in a uniform improvement in the material properties. Among the investigated formulations, 10 mol.% QAHAMA-10 provided the most favorable overall characteristics, including transparency, structural integrity, durability, and swelling behavior, while exhibiting effective antibacterial activity. However, this formulation also showed a reduced cell number of L929 fibroblasts.

The highest antibacterial activity against *S. aureus* was observed for the hydrogel containing 5 mol.% QAHAMA-10. Importantly, the hydrogel containing 2.5 mol.% QAHAMA-10 already exhibited effective antibacterial activity while showing the highest metabolic activity among the QAHAMA-10-containing formulations. Further increasing the QAHAMA-10 content therefore did not provide a clear overall benefit and was associated with progressively lower fibroblast cytocompatibility.

This preliminary study establishes a foundation for future research on QAHAMA-n-based antimicrobial hydrogels. Future studies should address the limitations of this preliminary evaluation by incorporating spectroscopic analyses, quantitative physical characterization, including mechanical testing, and quantitative microbiological assessment. Further optimization should focus on improving hydrogel structural integrity by adjusting the QAHAMA-10 content, as well as evaluating hydrogel durability over time. Systematic investigation of different QA substituents would also help to elucidate the effects of N-alkyl chain length on the physicochemical, antibacterial, and biological properties of the resulting hydrogels.

## Figures and Tables

**Table 1:** ^1^H NMR signal assignments for QAHAMA-10, as previously reported in our work [24].

Signal Symbol	Structure	Multiplicity	Number of Protons	Chemical Shift (ppm)
a	C**H_3_**-C=	s	3	1.92
b	=C**H_2_**	2 m	2	5.65 and 6.10
c	-O-C**H_2_**-CH_2_-N^+^	m	2	4.65
d	-O-CH_2_-C**H_2_**-N^+^	m	2	4.06
e	C**H_3_**-N^+^	s	3	3.39
f	HO-C**H_2_**-CH_2_-N^+^	m	2	3.81
g	HO-CH_2_-C**H_2_**-N^+^	m	2	4.13
h	-O**H**	bs	1	5.08
i	N^+^-C**H_2_**-CH_2_-(CH_2_)_7_-CH_3_	m	2	3.59
j	N^+^-CH_2_- C**H_2_**-(CH_2_)_7_-CH_3_	m	2	1.75
k	N^+^-CH_2_- CH_2_-(C**H_2_**)_7_-CH_3_	m	14	1.22–1.30
l	N^+^-CH_2_- CH_2_-(CH_2_)_7_-C**H_3_**	t	3	0.87

**Table 2:** ^13^C NMR signal assignments for QAHAMA-10, as previously reported in our work [24].

Signal Symbol	Structure	Chemical Shift (ppm)
a	=C-**C**H_3_	19
b	>**C**=	136
c	**C**H_2_=	127
d	**C**=O	166
e	CH_3_-(CH_2_)_8_-**C**H_2_-N^+^	56–64
	-**C**H_2_-N^+^	
	-**C**H_2_-O-	
f	**C**H_3_-N^+^	51
g	CH_3_-(**C**H_2_)_8_-CH_2_-N^+^	23–32
h	**C**H_3_-(CH_2_)_8_-CH_2_-N^+^	14

**Table 3:** Chemical composition of the prepared hydrogel formulations.

Sample Name	AA: QAHAMA-10 Molar Ratio	AA		QAHAMA-10		Bis-AA		KIO_4_: MONOMER Molar Ratio
		(mol.%) *	(wt.%) **	(mol.%) *	(wt.%) **	(mol.%) *	(wt.%) **	
H-0	100:0	99.04	97.88	0	0.00	0.96	2.12	1:796
H-I	97.5:2.5	96.52	85.49	2.47	12.61	1.01	1.90	1:3044
H-II	95:5	94.06	75.49	4.93	22.79	1.01	1.72	1:1523
H-III	90:10	89.11	60.16	9.88	38.39	1.01	1.45	1:761
H-IV	80:20	79.44	40.61	19.76	58.29	0.81	1.10	1:380
H-V	70:30	69.28	28.66	29.52	70.45	1.2	0.88	1:255

* The mol.% of a monomer refers to its molar percentage relative to the total moles of all monomers used in the formulation, calculated as mol.% = (moles of monomer/total moles of monomers) × 100. ** The wt.% refers to the weight percentage relative to the total mass of the monomers, calculated as wt.% = (mass of monomer/total mass of monomers) × 100, where the total monomers include AA + QAHAMA-10 + bis-AA.

**Table 4:** Summary of qualitative macroscopic properties of the hydrogels.

Sample Name	Optical Appearance	Structural Integrity During Swelling	Durability During Handling	Score
H-0 (control)	opaque/heterogeneous	disintegrated	completely unstable	1 (unstable)
H-I	opaque/heterogeneous	maintained integrity (no disintegration)	high fracture susceptibility	2 (weak)
H-II	opaque/heterogeneous	maintained integrity (no disintegration)	moderate brittleness	3 (moderate)
H-III	transparent/homogeneous	maintained integrity (no disintegration)	no visible cracks observed	5 (excellent)
H-IV	opaque/homogeneous	maintained integrity (no disintegration)	no visible cracks observed	4 (good)
H-V	opaque/homogeneous	maintained integrity (no disintegration)	increased brittleness	3 (moderate)

## Data Availability

The original contributions presented in this study are included in the article. Further inquiries can be directed to the corresponding author.

## Abbreviations

The following abbreviations are used in this manuscript:

AA	Acrylamide
BD	1-bromodecane
Bis-AA	N,N′-methylenenbisacrylamide
CDCl3	Deuterated chloroform
CFU/mL	Colony-forming units per milliliter
DMEM	Dulbecco’s Modified Eagle Medium
DS	Degree of swelling
*E. coli*	*Escherichia coli*
EDTA	Ethylenediaminetetraacetic acid
FBS	Fetal Bovine Serum
HAMA	N,N-(2-hydroxyethyl)methylaminoethyl methacrylate
H-0	Reference hydrogel
H-I	Hydrogel containing 2.5 mol.% QAHAMA-10
H-II	Hydrogel containing 5 mol.% QAHAMA-10
H-III	Hydrogel containing 10 mol.% QAHAMA-10
H-IV	Hydrogel containing 20 mol.% QAHAMA-10
H-V	Hydrogel containing 30 mol.% QAHAMA-10
IZD	Inhibition zone diameter
K_2_CO_3_	Potassium carbonate
MDEA	N-methyldiethanolamine
MgSO_4_	Magnesium sulfate
MMA	Methyl methacrylate
NaHCO_3_	Sodium bicarbonate
NMR	Nuclear magnetic resonance
PAM	Poly(acrylamide)
PES	polyethersulfone
PP	Potassium periodate
PTZ	Phenothiazine
*S. aureus*	*Staphylococcus aureus*
SD	Standard deviation
TCPS	Tissue culture polystyrene
TMS	Tetramethylsilane
UV-VIS	Ultraviolet–visible spectroscopy
QA	Quaternary group
QAHAMA-n	2-(methacryloyloxy)ethyl-2-hydroxyethylmethylalkylammonium bromides
QAHAMA-10	2-(methacryloyloxy)ethyl-2-decylhydroxyethylmethylammonium bromide
QAUDMA	Quaternary ammonium urethane dimethacrylates
